# Epigallocatechin-3-gallate reduces inflammation induced by calcium pyrophosphate crystals *in vitro*

**DOI:** 10.3389/fphar.2013.00051

**Published:** 2013-04-17

**Authors:** Francesca Oliviero, Paolo Sfriso, Anna Scanu, Ugo Fiocco, Paolo Spinella, Leonardo Punzi

**Affiliations:** ^1^Rheumatology Unit, Department of Medicine, University of PadovaPadova, Italy; ^2^Clinical Nutrition Unit, Department of Medicine, University of PadovaPadova, Italy

**Keywords:** osteoarthritis, epigallocatechin-3-gallate, inflammation, cytokines, chemokines

## Abstract

Although osteoarthritis (OA) is defined as a cartilage disease, synovitis involving mononuclear cell infiltration and overexpression of proinflammatory mediators is common in early and late OA. Calcium crystals deposition is thought to be a factor that likely contributes to synovial inflammation. In recent years, significant interest has emerged in the beneficial health effects attributed to the green tea polyphenols and in particular to epigallocatechin-3-gallate (EGCG). It has been demonstrated that some of the actions of EGCG are linked to its ability to interfere with cell membranes. The objective of this study was to evaluate the influence of EGCG in some inflammatory aspects of OA and whether EGCG is able to interfere with membrane organization. We assessed the effect of EGCG on the production of proinflammatory cytokines and chemokines released by human fibroblast-like synoviocytes (FLS) and THP-1 cells stimulated with calcium pyrophosphate (CPP) crystals in presence of methyl-β-cyclodextrin (MβCD), a cholesterol-removing agent that disturbs lipid raft structures. The chemotactic effect of culture supernatants was also evaluated. EGCG inhibited interleukin (IL)-1β, transforming growth factor beta, IL-8, and chemokine (C–C motif) ligand 2 (CCL2) release by stimulated FLS and/or THP-1 cells in a dose-dependent manner. Supernatants of CPP-stimulated cells induced the migration of neutrophils and mononuclear cells which decreased in a dose-dependent manner in the presence of EGCG. EGCG increased cell viability when added to THP-1 cells treated with MβCD. Furthermore, MβCD enhanced the inflammatory response to CPP crystals increasing IL-8 and CCL2 secretion which was inhibited by EGCG in a dose-dependent manner. This study showed that EGCG is able to reduce the inflammatory response induced by CPP crystals *in vitro*. The identification of EGCG as dietary supplement capable of affording protection or modulating the inflammatory response to CPP crystals may have important implications in the prevention and treatment of OA and crystal-related arthropathies.

## INTRODUCTION

Although osteoarthritis (OA) is defined as a cartilage disease, synovitis involving mononuclear cell infiltration and overexpression of proinflammatory mediators is common in early and late OA (Benito et al., 2005; Punzi et al., 2005).

Calcium crystals deposition is thought to be a factor that likely contributes to synovial membrane inflammation (Jaovisidha and Rosenthal, 2002).

Calcium pyrophosphate (CPP) dihydrate crystals and basic calcium phosphate crystals are often present in synovial fluid from patients with OA. They have been demonstrated in up to 60% of knee OA effusions (Gibilisco et al., 2009) with a prevalence of about 22% for CPP (Oliviero et al., 2012b), and in 100% of knee and hip cartilage of patients with end stage of OA undergoing replacement (Fuerst et al., 2009). These crystals have a still undefined role in OA inflammation but, whether primary or secondary to tissue degeneration they may accelerate the osteoarthritic process (Nalbant et al., 2003).

Green tea (*Camellia sinensis*) is one of the most commonly consumed beverages in the world and is a rich source of polyphenols known as catechins including epigallocatechin-3-gallate (EGCG) which constitutes up to 63% of total catechins (Manning and Roberts, 2003).

Epigallocatechin-3-gallate has been shown to exhibit protective effects on a number of clinical conditions, including stroke and cerebral hemorrhage, cardiovascular and liver diseases, bacterial infections, cancer, atherosclerosis, and autoimmune diseases (Mak, 2012). Extensive studies in the past decade have also verified the cartilage-preserving and chondroprotective action of EGCG (Ahmed et al., 2006; Morinobu et al., 2008).

It has been demonstrated that some of the actions of EGCG are linked to its binding to the cell surface and the interaction with the plasma membrane microdomains referred to as “lipid rafts” (Duhon et al., 2010). Furthermore the antiproliferative effect and antioxidant capacity of EGCG seem to be associated to its ability to modify lipid bilayers, the membrane fluidity and to its stabilizing effect on the cell membranes (Tsuchiya et al., 2008; Margina et al., 2012).

The objective of this study was to evaluate the influence of EGCG in some inflammatory aspects of OA and whether EGCG is able to interfere with lipid membrane organization.

To this aim, we assessed the effect of EGCG on the production of proinflammatory cytokines and chemokines released by human fibroblast-like synoviocytes (FLS) and THP-1 cells stimulated with CPP crystals in the presence of methyl-β-cyclodextrin (MβCD), a cholesterol-removing agent that disturbs raft structure.

## MATERIALS AND METHODS

### FLS ISOLATION AND TREATMENT

Synovial tissue specimens were obtained from OA patients undergoing surgical joint replacement. FLS were isolated from tissue explants, as previous described (Scanu et al., 2007) and grown in Dulbecco’s modified Eagle’s medium (DMEM) containing 10% heat-inactivated fetal calf serum (FCS), 50 μg/ml streptomycin, 50 U/ml penicillin, and 2 mmol/l glutamine. All experiments were carried out with passage 4 through 8. FLS were CD90^+^, CD55^+^, and were positive for prolyl-4-hydroxylase, as demonstrated by immunocytochemical staining with specific antibodies (Chemicon International). FLS were seeded in 96-well culture plates at a density of 1 × 10^4^ cells/well. Cells were allowed to adhere for 24 h, and then the medium was exchanged for a medium supplemented with 2% FCS, CPP crystals 0.025 mg/ml and EGCG 1, 5, or 10 μM. A mother solution of EGCG 10 mM was prepared in phosphate buffered saline (PBS) solution and diluted in the medium at the time of experiment while crystals were suspended directly in the medium.

### THP-1 CELL CULTURE AND TREATMENT

The human leukemic monocytic cell line THP-1, was obtained from the American Type Culture Collection (Rockville, MD, USA). Cells were cultured in Roswell Park Memorial Institute (RPMI) 1640 medium supplemented with 10% FCS. In some experiments, cells were primed for 3 h with phorbol 12-myristate 13-acetate (PMA; Sigma-Aldrich) at 300 ng/ml and reincubated overnight with fresh medium supplemented with 10% FCS. Cells were then treated with CPP (final concentration 0.025 mg/ml; InvivoGen) for 24 h in presence or absence of EGCG (10, 50 μM) and 2% FCS. Cells incubated with medium alone served as controls.

In some experiments, before stimulation with crystals FLS and THP-1 were pretreated for 1 h with EGCG at the indicated concentrations or for 45 min with 10 mM MβCD (Sigma-Aldrich) prepared from a 500 ng/ml PBS mother solution.

To exclude a contribution of endotoxin contamination, 10 μg/ml of polymyxin B (Sigma-Aldrich) was included in all the stimulation assays.

### CYTOKINE DETERMINATION

The levels of interleukin (IL)-1β, IL-8, chemokine (C–C motif) ligand 2 (CCL2) and transforming growth factor beta (TGFβ) were determined in the culture supernatants by enzyme-linked immunosorbent assay (eBioscience).

### MTT ASSAY

To measure cell viability and proliferation the colorimetric MTT (3-(4,5-dimethylthiazol-2-yl)-2,5-diphenyltetrazolium bromide) mitochondrial activity assay (Chemicon) was used.

Cells were cultured in a 96-well plate at 37°C, and exposed to varying concentrations of crystals, EGCG for 24 h. Cells treated with medium only served as a negative control group. After removing the supernatant cells were incubated with the MTT solution for 4 h and the resultant formazan crystals were dissolved in dimethyl sulfoxide and the absorbance intensity measured at 570 nm. The cell viability (%) was expressed as a percentage relative to the untreated control cells.

### CHEMOTAXIS ASSAY

After inform consent, mononuclear and polymorphonuclear (PMN) cells were isolated from peripheral blood from healthy volunteers by density gradient centrifugation with Histopaque 1077 (Sigma-Aldrich).

The chemotactic effect of culture supernatants of cells exposed to CPP crystals and EGCG was assessed by using a 48-well modified Boyden chamber (AC48; NeuroProbe) as previously described (Scanu et al., 2010). In brief, 28 μl aliquots of culture supernatants were loaded in the bottom chamber, and mononuclear or PMN cells were added to the top chamber. RPMI 1640 was used as a negative control, while 10 ng/ml CCL2 or IL-8 (RayBiotech) were used as a positive control. A polyvinylpyrrolidone-free polycarbonate 8-mm membrane with 5-μm or 3-μm pores, pretreated with 10 μg/ml fibronectin, was placed between the chambers. Fifty-microliter aliquots of mononuclear cells (1 × 10^6^ cells/ml) or PMN cells (3 × 10^6^ cells/ml) resuspended in RPMI 1640 were added to the top wells.

Chambers were incubated at 37°C with 5% CO_2_ for 2 h. The membrane was then removed, washed with PBS on the upper side, fixed, and stained with DiffQuik (Baxter Scientific). Cells were counted microscopically at ×1,000 magnification and expressed as total number of cells per membrane. All assays were performed in duplicate.

### STATISTICS

Results are presented as mean ± SD of *n* experiments performed in triplicate. Statistical differences were determined by non-parametric Mann–Whitney test, taking a *p*-value < 0.05 as significant.

## RESULTS

### EGCG INHIBITS CPP CRYSTAL-INDUCED CYTOKINE AND CHEMOKINE PRODUCTION IN FLS AND THP-1 CELLS

**FIGURE 1:**
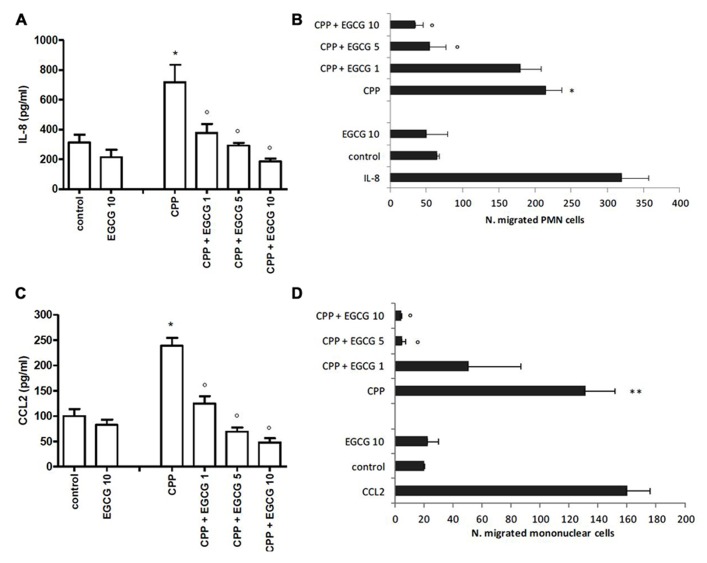
**Effect of EGCG on IL-8 and CCL2 production by FLS stimulated with CPP crystals (A,B)**. Effect of culture supernatants on the migration of PMN and mononuclear cells **(C,D)**. The values are expressed as the mean ± SD; CPP 0.025 mg/ml; EGCG expressed as micromolar. Data are representative of one of three independent experiments ± SD of three replicates. *p < 0.05, **p < 0.01 vs control; °*p* < 0.05 vs CPP.

In the absence of stimulus, FLS released low but significant levels of both IL-8 and CCL2 in culture supernatants (**Figures 1A, B**).

Calcium pyrophosphate crystals stimulation of FLS induced an increased release of IL-8 and CCL2 over the basal concentration. This effect was suppressed by EGCG which inhibited chemokine levels in a dose-dependent manner. EGCG slightly affected also the basal release of these chemokines (**Figures 1A, B**).

Supernatants of CPP-stimulated cells induced the migration of PMN cells (about sixfold over the control) and mononuclear cells (about fourfold over the control) which decreased in a dose-dependent manner in the presence of EGCG, with the basal migration effect restored with EGCG 5 μM (**Figures 1C, D**).

**FIGURE 2:**
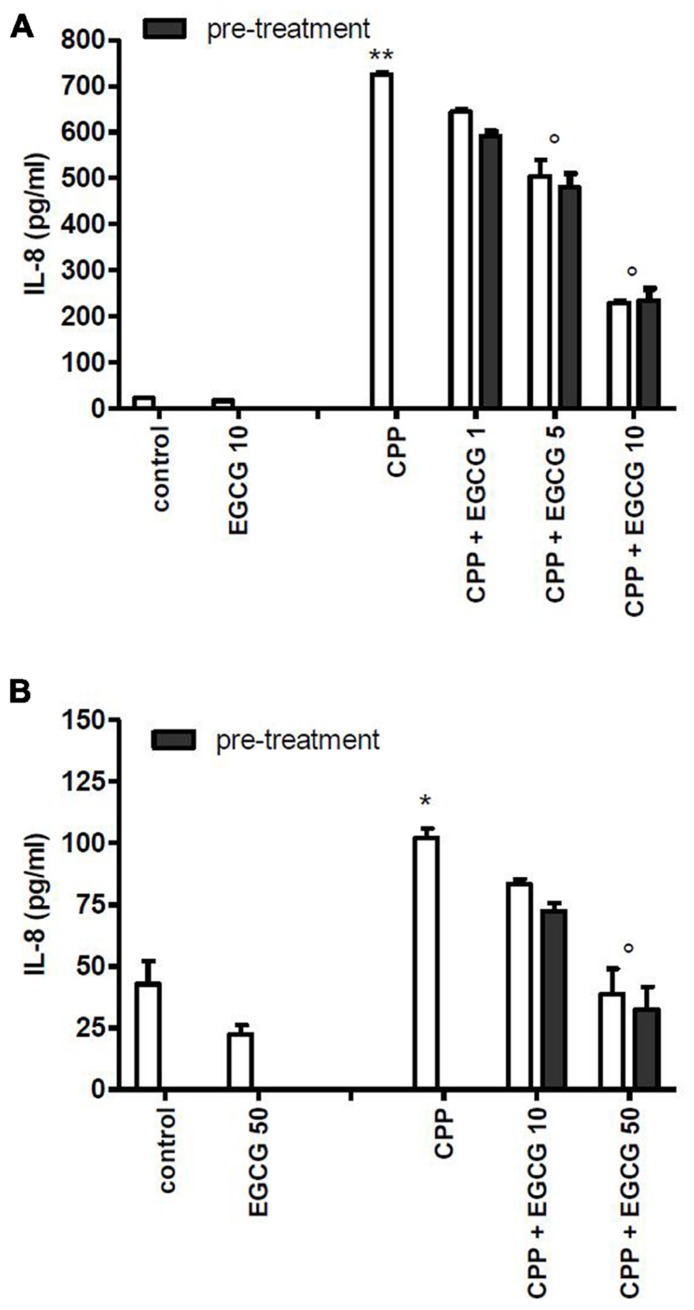
**Effect of 1 h pretreatment of cells with EGCG before CPP crystal stimulation on IL-8 production.** Results from FLS **(A)** and THP-1 **(B)** cells. The values are expressed as the mean ± SD; CPP 0.025 mg/ml; EGCG expressed as micromolar. Data are representative of one of three independent experiments ± SD of three replicates. *p < 0.05, **p < 0.01 vs control; °*p* < 0.05 vs CPP.

To evaluate whether the effect of EGCG was mediated by a direct action on cell, FLS and THP-1 cells were pretreated with EGCG and washed before crystal stimulation. EGCG at 1, 5, and 10 μM reduced chemokine levels on culture medium also in pretreated cells (**Figure 2**).

**FIGURE 3:**
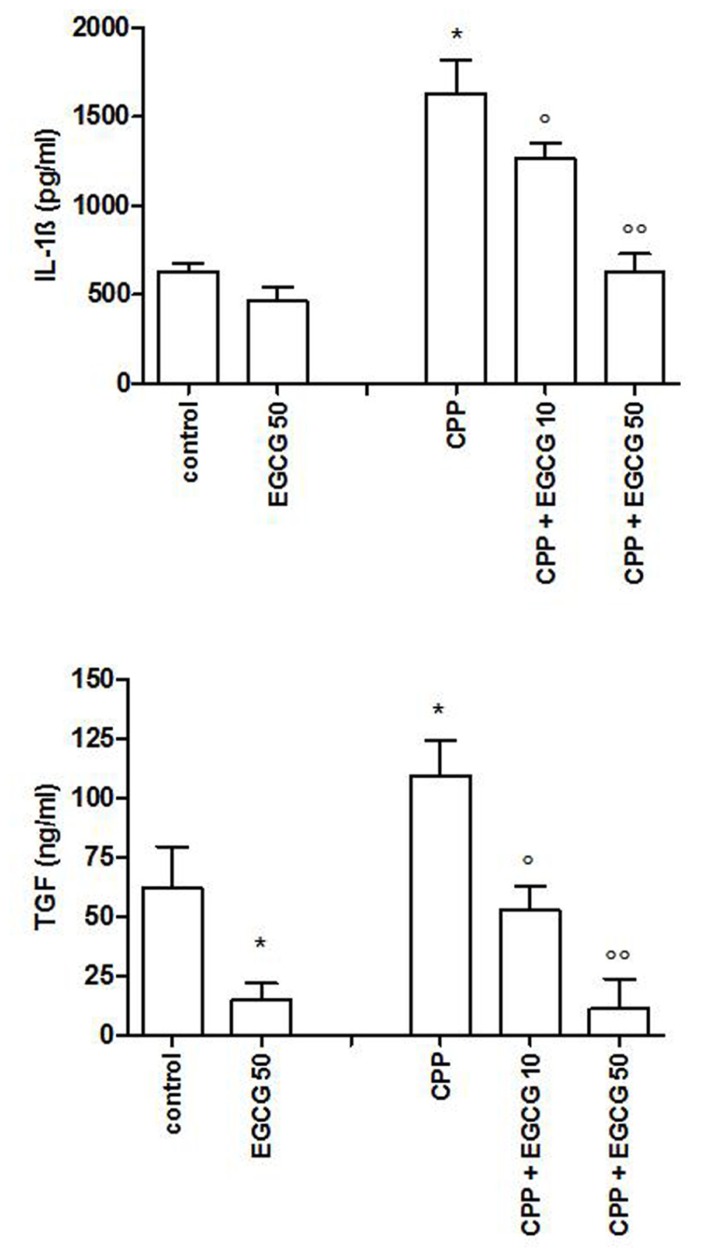
**IL-1β and TGFβ levels after a pretreatment of 3 h ofTHP-1 cells with PMA and stimulation with CPP crystals.** The values are expressed as the mean ± SD; CPP 0.025 mg/ml; PMA 300 ng/ml; EGCG expressed as micromolar. Data are representative of one of three independent experiments ± SD of three replicates. *p < 0.05 vs control; °*p* < 0.05, °°*p* < 0.01 vs CPP.

As CPP crystals alone are not able to induce IL-1β and TGFβ production, THP-1 cells were primed with PMA 300 ng/ml for 3 h, followed by an overnight medium renewal and crystal stimulation. THP-1 cells showed a marked reduction in IL-1β and TGFβ production after EGCG treatment (**Figure 3**).

Epigallocatechin-3-gallate alone showed a trend toward reduction of IL-8, CCL2, IL-1β, and TGFβ release from non-treated cell. As regards the overall effect of EGCG, it has been observed by others that EGCG added to DMEM underwent rapid oxidation to generate H_2_O_2_ (Long and Halliwell, 2009) but that in presence of pyruvate in the medium (final concentration 1 mM) much less H_2_O_2_ was detected. In particular, for concentrations of EGCG up to 100 μM, almost no H_2_O_2_ was detected at all. In our experiments, we used EGCG at concentration <100 μM and we can therefore exclude that the effect of EGCG against CPP stimulation is an artifact due to oxidation products.

### EGCG INCREASES CELL VIABILITY

To assess whether the effect of EGCG was associated with modifications on membrane organization THP-1 cells were treated 45 min with 10 mM MβCD before CPP crystal stimulation and cell activity was examined.

**FIGURE 4:**
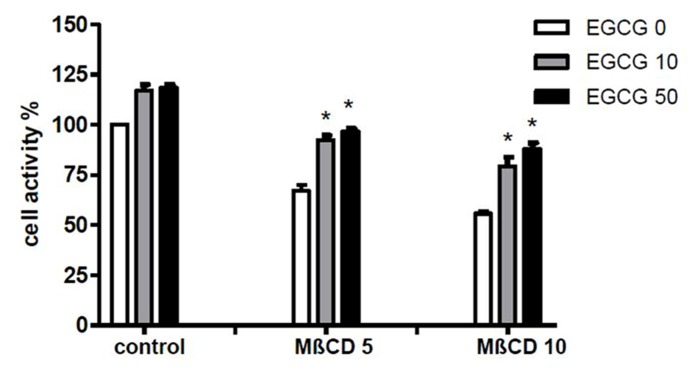
**Effect of EGCG on cell damage induced by MβCD.** Cells were preincubated 45 min with MβCD at the indicated concentrations and treated with EGCG. Cell activity was then evaluated after 24 h. The values are expressed as the mean ± SD; MβCD expressed as millimolar; EGCG expressed as micromolar. Data are representative of one of three independent experiments ± SD of three replicates. *p < 0.05 vs EGCG 0.

The results demonstrated that EGCG at 10 and 50 μM increased cell viability in THP-1 cells both untreated and treated with different concentrations of MβCD (**Figure 4**).

**FIGURE 5:**
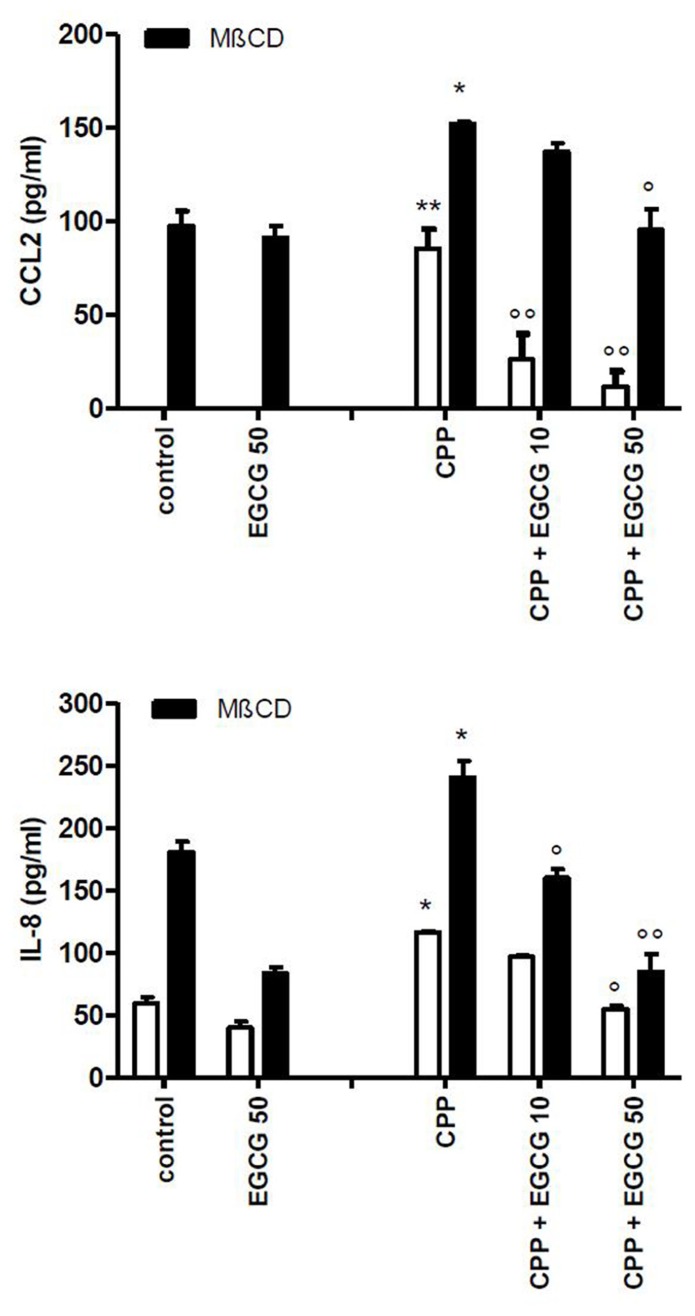
**Effect of EGCG on IL-8 and CCL2 production byTHP-1 stimulated with CPP crystals after a pretreatment of 45 min with MβCD.** The values are expressed as the mean ± SD; CPP 0.025 mg/ml; MβCD expressed as millimolar; EGCG expressed as micromolar. Data are representative of one of three independent experiments ± SD of three replicates. *p < 0.05, **p < 0.01 vs control; °*p* < 0.05, °°*p* < 0.01 vs CPP.

The treatment of cells with MβCD significantly increased the release of IL-8 and CCL2 into the medium with respect to control conditions. Furthermore the inhibitory action of EGCG on MβCD-enhanced inflammatory response to CPP crystals (**Figure 5**) suggests that the effect of EGCG is independent from lipid membrane organization.

## DISCUSSION

This study showed that EGCG, the main catechin of green tea, is able to reduce cytokine release in FLS and THP-1 cells. We have found a basal release of CCL2 and IL-8 in culture supernatants of both untreated FLS and THP-1 cells which enhanced in presence of CPP crystals. EGCG slightly affected the basal release of these chemokines but reduced CPP crystal-induced inflammation into a baseline level with an appropriate dosage.

While calcium crystals in OA are associated with a more severe disease, in pseudogout they can cause powerful inflammatory reactions comparable to those triggered by monosodium urate crystals. The concentrations of CPP crystals used in this study are similar to those observed in synovial fluids collected during intercritical attacks of pseudogout and therefore reproduce a pathophysiological condition (Swan et al., 1995).

It is known that CPP crystals activate specific signaling pathway leading to the production of inflammatory cytokines and chemokines in the synovial compartment (Busso and Ea, 2012).

Activated resident FLS are known to be central mediators of crystal-induced inflammation through the production of cytokines and chemokines that mediate the recruitment and activation of leukocytes. We have previously demonstrated the presence of intracellular stores of CCL2 in FLS which might participate in the rapid response of joint cells to crystals, attracting monocytes/macrophages into the tissue in an attempt to eliminate the inflammatory agent rapidly (Scanu et al., 2010).

Through inflammasome assembly, CPP crystals are able to activate caspase-1 and subsequently the release of IL-1β in the extracellular space (Gombault et al., 2012). But a second stimulus is needed to prime cells to generate IL-1β *in vivo*. It has been hypothesized that serum protein (Oliviero et al., 2012a) and Toll-like receptor ligands such as free fatty acids (Mylona et al., 2012) synergize with crystals on IL-1β induction. In this study, we used the protein kinase C activator PMA which influences cell differentiation and stimulate monocyte functions including phagocytosis.

After cell priming, we observed an increase of IL-1β and TGFβ production by CPP crystals which was inhibited by EGCG. TGFβ is involved in crystal formation through the generation of extracellular inorganic pyrophosphate (Olmez et al., 1994). Its expression is strongly associated to hypertrophic chondrocytes around calcium crystal deposits and its action is antagonized by IL-1β (Lotz et al., 1995). We had previously found increased TGFβ level in OA synovial fluid positive to CPP crystals with respect to OA synovial fluid without crystals (Punzi et al., 2003) but little is known on the direct effect of CPP on TGFβ production and if the latter may be linked on IL-1β release. 

Along with the anti-inflammatory effect of EGCG on cytokine and chemokine concentrations, we also observed an anti-chemokinetic effect on human neutrophil and mononuclear cells induced by culture supernatants of cells exposed to crystals and EGCG.

As cell migration play a significant role on the amplification of the inflammatory response to pathogenetic crystals in terms of synovial cell and endothelial activation (Liu-Bryan and Lioté, 2005), this also represents an important results of this work.

Another important finding is that the anti-inflammatory capacity of EGCG could be due to its protective and direct effect on the cell membranes.

It has been observed that green tea catechins have a stabilizing effect on the cell membranes reducing fluidity of lipid bilayers (Tsuchiya, 1999) and increasing membrane anisotropy and polarization (Margina et al., 2012).

Although our results does not elucidate the exact mechanism of action of EGCG, we have shown that the pretreatment of cells with EGCG is also effective in inhibiting chemokine release. Furthermore, EGCG was able to increase cell viability also after the destruction of specific cell membrane microdomains by MβCD.

However, our results showed that MβCD treatment does not affect EGCG effect on the inflammatory response to CPP crystals suggesting an independent role of EGCG from lipid rafts.

It has been demonstrated that only a small percentage of the ingested catechins appears in the blood. After drinking the equivalent of ~2 cups of tea, the mean peak plasma EGCG level has been shown to be <1 μM (Lee et al., 2002). Although these values are lower than those used in *in vitro* experiments with EGCG, an effective concentration of this catechin could be achieved by dietary supplementation.

In conclusion, the use of EGCG in our *in vitro* model of CPP crystal-induced inflammation lead to a significant inhibition of the inflammatory response.

Osteoarthritis, besides CPP crystal-related arthropathies, continue to be difficult disorders to treat, as there is no cure as such and current treatments focus mainly on relieving pain and maintaining joint function. None of the therapeutic approaches appear to be able to spare cartilage from the ongoing degenerative process of OA and from synovitis in pseudogout (Dingle, 1991; Schlesinger et al., 2009). In this context the identification of EGCG as substance capable of affording protection or modulating the inflammatory response to CPP crystals may have important therapeutic implications. Further studies elucidating the role of EGCG dietary supplementation are needed.

## Conflict of Interest Statement

The authors declare that the research was conducted in the absence of any commercial or financial relationships that could be construed as a potential conflict of interest.
